# Resident scholary work in the community hospital setting

**DOI:** 10.3402/jchimp.v1i3.8486

**Published:** 2011-10-17

**Authors:** Robert P. Ferguson

The primary reason to develop the Journal of Community Hospital Internal Medicine Perspectives (www.JCHIMP.org) was to focus on and encourage the scholarly work of faculty and physicians in training (students, residents, and fellows) in community teaching hospitals. Our secondary focus was based on the belief that the presence of quality research is of itself a goal to strive for. Our third focus was to encourage compliance with the stated goals of the Internal Medicine Residency Review Committee regarding residency scholarly work.

Let's start with the latter. The Resident Review Committee (RRC) scholarly activities section reads as follows: Section IV.B.1 ‘The curriculum must advance residents’ knowledge of the basic principles of research.’ Section IV.B.2 also reads: ‘Residents should participate in scholarly activity.’ Section IV.B.3 further states: (1) ‘The sponsoring institution and program should allow adequate educational resources to facilitate resident involvement in scholarly activity.’

Why is scholarly activity a requirement? It is a raised bar that has great merit. Scholarship is considered a major component of good medicine and is the basis of medical intervention and clinical excellence. Scholarship breeds attitudes that benefit trainees. Scholarship enhances faculty development. Scholarship employs scientific discipline. Scholarship requires discipline and attention to detail. Participation in research studies improves accurate observation skills. A major part of scholarship is peer review; the ultimate test of putting your scholarly work under expert scrutiny. Scholarly work emphasizes the evidence-based approach.

Many residency programs in the United States are linked to medical schools, often physically in the same location. The mission of many of these institutions is research; and to ensure success, the faculty is recruited and revenues are set aside. Many of these institutions also have long histories affiliated with sponsoring organizations such as the National Institutes of Health and private sector sponsorship.

Although scholarship is a RRC requirement, there have been relatively few publications of research curricula for internal medicine residencies. One of the recently published exceptions was the Wake Forest University Baptist Medical Center's Resident Research Program developed in 2007. The program was published earlier this year (2). Wake Forest has greater than 150 full-time faculty in its department, including dedicated research faculty. They have a long-standing relationship of cooperation with the National Institutes of Health, dedicated physician scientists, and clinical investigators. Their categorical resident program includes a curriculum that has 3 to 5 months of dedicated research, with at least 1 month each year. They also have a chief resident for research and a faculty research director. The program has many faculty research mentors, as well. Outcomes from this program have been excellent. Papers published and presentations nationally and regionally have increased dramatically since 2007. Of course, the necessary funding support is there, although the authors did not offer details.

Unfortunately, not all residency programs have access to such wonderful research support, and this particularly applies to community hospitals that make up more than half the sponsored internal medicine programs in the United States. Aside from lack of funding, they have other major obstacles to overcome compared to most university programs.

1. The numbers of residents generally are fewer, which lead to less flexibility in scheduling. I doubt there are many community-based programs that include 3 to 5 months of research for each resident.
2. Dedicated full-time research faculty tend to be fewer with much mentoring being done by volunteer physicians.
3. Laboratories and supporting departments such as radiology/pathology usually do not have a research agenda.
4. Often, there is a lack of research tradition.

However, even with such hurdles, good scholarly work can be accomplished in the community hospital setting and should be. In some respects, certain types of research in the community hospital setting may be more ‘real world’ and more easily applicable to current clinical practice.

To contrast the Wake Forests curriculum, the curricular program in research at Union Memorial Hospital Internal Medicine began in the 1990s through a number of steps. We established a faculty/residency research committee that included a medical writer, (we borrowed her from orthopedics!). Our head librarian was included, and this committee was chaired by an experienced subspecialist with a research background. This group measures each resident's scholarly activity three times a year.

Also, during the 1990s, we established the Union Memorial Summer Research Student Program. We advertised for rising second-year students, the majority of whom came from the University of Maryland. A summer session was optimal, since after the first year, a medical student at the University of Maryland only has free time available in the summer. We involve projects that are developed by the research committee and mentors. The summer program is augmented through student-focused research conferences that are held at the beginning of each session. We set aside approximately $20,000 a year for this student-based program. The investment has been well worth it. When the summer students leave, a faculty/resident combination continues the project through the next summer for a new student class, if necessary.

Creating the research committee and student programs was essential. Yet, electronic medical records’ (EMR) importance to scholarship cannot be overstated and in the period following the phase in of EMR at our institution over the last 10 years, our research output has accelerated. The productivity has been excellent. As of July 2011, our student resident bibliography includes 24 peer-reviewed papers and 64 abstracts (3). Most of these studies started by observing problems in a patient at morning report.

Our blossoming tradition of research blends established scholarship avenues with innovative approaches to foster student and resident participation. For example, Maryland has the luxury of a very active American College of Physicians (ACP) chapter, and our residents are encouraged to participate in the ACP Associates (resident members) research competition every spring. In addition, in the last 5 years, we have had a similar experience at the student level in teaming up with the ACP Washington, DC, chapter. This hospital also sponsors an ‘Author's Day’ for all residents,’ posters, and regional presentations. It seems that spreading the word through an institution is the first step in developing a culture of research. This can be a challenge. It is important to have the administration of your institution sympathetic. Once the administration makes the connection between scholarly work and good medical care, they see the inherent value of scholarly work that tends to make them more generous.

Presenting orally or with a poster is an important component of academic training. All residents are encouraged to do a scholarly project at our program but are not required if in their previous career, they were primarily involved with research. This is not a small number; approximately one in three of our categorical residents come from a research background.

The basics of a community-based scholarly program include core faculty with experience at research, interest, and protected time. One needs additional resources specifically dedicated to student and resident support for the scholarly projects. A good EMR is important because most of the studies in this setting will be observational. Observational studies and case reports are important components of what we do and reflect the real world. They are practical, doable, and relevant, although there are obvious limitations in study design. Observational studies are the bedrock of practice-based learning.

Case reports can be extremely interesting and scholarly and do not normally require dedicated blocks of time to complete during training. Developing a culture where a clinical vignette is considered of value is the key thing. Dan Reisenburg, 25 years ago, said ‘Send us such observations. We appreciate your first perspective on an important problem.’ The will to complete a good case report is the most important factor (4).

I want to comment about the JCHIMP that you are reading. In the summer of 2010, we developed the idea primarily because when we looked, there were not a lot of peer-reviewed publications that seemed interested in the type of scholarship, observational studies, case reports, and clinical perspectives that are the mainstay of the scholarly work at community hospitals. In fact, we could find no other online journal that included community hospital in the title. We are trying to fill that niche. We are committed to community hospital scholarly work as our first priority. Just 1 year later, we are now publishing our third issue of JCHIMP and have expanded. We have established the following statistics as of August 10, 2011 (Table 1).

**Table 1:** JCHIMP statistics August 10, 2011

Total visits – 3,274
Unique visitors – 1,513
Registered readers – 215
Authors – 66
Manuscripts – 27
Published manuscripts – 20
Reviewers – 120
Requested to review – 67
Completed review request – 50
75% of Requested reviews get completed

In this third issue, we will have six papers (not including this one). Timothy Woodlock and colleagues have studied the approach to lung cancer in the very elderly in community hospitals (5) Carla McWilliams and colleagues have shown that vitamin D levels can predict certain outcomes for elderly inpatients (6). Roger Leonard is a former invasive cardiologist (7). He reviews the current controversial state of affairs in interventional cardiology. Sapna Kuehl and Richard Pomerantz have made two important contributions to our Medical Education section (8, 9). Sapna has put together a cogent piece on doctor–patient communication. Richard has provided the perspective of a former cardiology fellowship director on how best to pursue a fellowship, timely, in light of the NRMP changes being implemented as we speak. Marc Mugmon's ECG column shows that, as in everything else in electrocardiography, hyperkalemic changes follow a progression (10).

In issue #4, we intend to add two new features – Clinical Images and History of Medicine. Please feel free to contribute your ideas, writings, cases, reviews, and research to the Journal of Community Hospital Internal Medicine Perspectives. We won't turn down your donations either. It is easy to donate on the website and it is fully tax deductible.

*Robert P. Ferguson, MD* Editor
